# Comparative Prognostic Performance of Nutritional and Inflammatory Indices in Diffuse Large B-Cell Lymphoma

**DOI:** 10.3390/jcm15124703

**Published:** 2026-06-17

**Authors:** Tahir Alper Cinli, Gökhan Burul, Hasan Göze, Mesut Ayer, Istemi Serin

**Affiliations:** Department of Hematology, Başakşehir Çam and Sakura City Hospital, 34480 Istanbul, Turkey; drburulgokhan@gmail.com (G.B.); hasangoze@hotmail.com (H.G.); mesutayerdr@hotmail.com (M.A.); serinistemi@hotmail.com (I.S.)

**Keywords:** diffuse large B-cell lymphoma, prognostic nutritional index, HALP score, geriatric nutritional risk index, overall survival, prognosis

## Abstract

**Background**: Diffuse large B-cell lymphoma (DLBCL) is the most common aggressive non-Hodgkin lymphoma. Despite advances in immunochemotherapy, approximately 30–40% of patients experience relapsed or refractory disease. Nutritional and inflammatory status, reflected by composite indices, may independently influence clinical outcomes. However, the prognostic value of the Prognostic Nutritional Index (PNI), Geriatric Nutritional Risk Index (GNRI), and Hemoglobin-Albumin-Lymphocyte-Platelet (HALP) score has not been well established in DLBCL patients treated with rituximab-based regimens. **Methods**: We retrospectively analyzed 192 patients with newly diagnosed DLBCL who received at least three cycles of R-CHOP or R-EPOCH at Başakşehir Çam and Sakura City Hospital between January 2020 and January 2026. Receiver operating characteristic (ROC) curve analysis was performed to determine optimal cutoff values. Kaplan–Meier analysis with log-rank testing and univariable/multivariable Cox proportional hazards regression analyses were used to evaluate the prognostic impact of the PNI, GNRI, and HALP on overall survival (OS) and progression-free survival (PFS). **Results**: Among the six indices evaluated (PNI, GNRI, HALP, SII, ALI, and CAR), the PNI demonstrated the highest discriminatory ability for OS (AUC = 0.734, *p* = 0.001), followed by the HALP (AUC = 0.671, *p* = 0.020) and GNRI (AUC = 0.668, *p* = 0.022). The optimal cutoff values were ≤46.45 for the PNI, ≤46.91 for the GNRI, and ≤223.95 for HALP. Low values of all three indices were significantly associated with elevated LDH levels, advanced Ann Arbor stage, and higher IPI category. Kaplan–Meier analysis demonstrated significantly inferior OS in the low PNI (52.8 ± 2.6 vs. 67.1 ± 1.2 months, *p* = 0.001), low GNRI (49.5 ± 3.1 vs. 66.0 ± 1.4 months, *p* = 0.001), and low HALP (58.8 ± 2.8 vs. 64.9 ± 1.2 months, *p* = 0.005) groups. In separate multivariable Cox models adjusted for sex and IPI, the PNI (HR = 0.216, *p* = 0.009), HALP (HR = 0.276, *p* = 0.031), and GNRI (HR = 0.294, *p* = 0.011) remained independently associated with OS. No significant association was observed between these indices and PFS. **Conclusions**: The PNI, GNRI, and HALP are independent prognostic markers in patients with DLBCL treated with rituximab-based regimens. These readily available and inexpensive baseline indices may complement the IPI in identifying patients at higher risk of adverse outcomes and support risk stratification at diagnosis.

## 1. Introduction

Diffuse large B-cell lymphoma (DLBCL) is the most common histological subtype of non-Hodgkin lymphoma (NHL). It represents approximately 30–40% of newly diagnosed cases worldwide [1]. The addition of rituximab to standard chemotherapy with cyclophosphamide, doxorubicin, vincristine, and prednisone (CHOP) has significantly improved outcomes. More than half of previously untreated patients have achieved durable remission with this treatment [2,3]. However, approximately 30–40% of patients progress to relapsed or refractory disease [4]. For this reason, accurate risk assessment at diagnosis is essential for treatment decisions. The International Prognostic Index (IPI), which was developed before the introduction of rituximab, remains the most widely used clinical prognostic tool for DLBCL [5]. Later improvements, including the Revised IPI (R-IPI) and NCCN-IPI, have enhanced risk stratification in the rituximab era [6,7]. Nevertheless, these indices are based solely on clinical and laboratory parameters and do not account for nutritional or inflammatory host responses, which are increasingly recognized as key determinants of tumor behavior and treatment tolerance [8].

The tumor microenvironment is characterized by complex interactions among malignant B cells, immune effector cells, and inflammatory mediators. Peripheral blood cell counts provide insight into this microenvironment. The neutrophil-to-lymphocyte ratio (NLR) and platelet-to-lymphocyte ratio (PLR) correlate with clinical outcomes in DLBCL [9,10]. Recently, composite indices that incorporate nutritional status, such as the Prognostic Nutritional Index (PNI), Geriatric Nutritional Risk Index (GNRI), and Hemoglobin-Albumin-Lymphocyte-Platelet (HALP) score, have gained attention as markers of inflammatory and immunonutritional status [11,12,13].

Originally proposed by Onodera et al. as a preoperative nutritional assessment tool, the PNI incorporates serum albumin and peripheral lymphocyte count [14]. It has shown prognostic value in various solid tumors and, more recently, in hematologic malignancies [15,16]. The GNRI, which is derived from serum albumin and the ratio of current to ideal body weight, was initially developed to assess malnutrition risk in hospitalized older adults and has subsequently been validated as a prognostic marker in cancer [17,18,19]. HALP, which combines hemoglobin, albumin, lymphocyte, and platelet values, has also emerged as an independent prognostic factor in several solid tumors [20,21]. However, data on HALP in DLBCL remain limited.

Given the multiparametric nature of these indices and the limited comparative data in DLBCL, we aimed to compare the prognostic performance of six nutritional and inflammatory indices using ROC analysis: the Prognostic Nutritional Index (PNI), Geriatric Nutritional Risk Index (GNRI), Hemoglobin-Albumin-Lymphocyte-Platelet (HALP) score, Systemic Immune-Inflammation Index (SII), Advanced Lung Cancer Inflammation Index (ALI), and C-reactive protein-to-albumin ratio (CAR). We also assessed the association of the three best-performing indices with clinicopathological characteristics and evaluated their independent prognostic significance for overall survival (OS) and progression-free survival (PFS) in a contemporary cohort of DLBCL patients treated with rituximab-based regimens at a tertiary referral center.

## 2. Materials and Methods

### 2.1. Study Population

A total of 255 patients diagnosed with DLBCL between January 2020 and January 2026 at Başakşehir Çam and Sakura City Hospital were retrospectively screened for eligibility. Sixty-three patients were excluded because of missing pre-treatment laboratory data or because they did not receive rituximab in combination with CHOP or CHOEP chemotherapy. In the final analysis, 192 patients were included. The inclusion criteria were: (1) a diagnosis of DLBCL according to the 2016 World Health Organization (WHO) Classification of Tumours of Haematopoietic and Lymphoid Tissues; (2) treatment with at least three cycles of R-CHOP or R-EPOCH; and (3) availability of complete laboratory data obtained within one week before treatment initiation. Patients were excluded if they had active infection at diagnosis, HIV infection, transformed indolent lymphoma, primary CNS B-cell lymphoma, primary mediastinal large B-cell lymphoma, high-grade B-cell lymphoma, intravascular large B-cell lymphoma, a concomitant secondary malignancy, or were younger than 18 years of age or pregnant. To exclude active infection, all patients underwent clinical and laboratory evaluation, including CRP, procalcitonin, and computed tomography of the affected area when clinically indicated. No pre-specified sample size calculation was performed. Instead, all consecutive patients diagnosed with DLBCL between January 2020 and January 2026 at our institution were retrospectively screened, and all eligible patients were included in the final analysis.

### 2.2. Ethical Statement

Ethics approval for this study was obtained from the Başakşehir Çam and Sakura City Hospital Ethics Committee (decision no. 263; 6 May 2026). The study was conducted in accordance with the principles of the Declaration of Helsinki. Given the retrospective design and the use of anonymized patient data, the requirement for informed consent was waived by the Ethics Committee.

### 2.3. Statistical Analysis

The normality of continuous variables was assessed using the Shapiro–Wilk test. Normally distributed variables were presented as mean ± standard deviation (SD), whereas non-normally distributed variables were presented as median (interquartile range [IQR]). Categorical variables were presented as frequencies and percentages and were compared using the Pearson chi-square test or Fisher’s exact test, as appropriate.

Receiver operating characteristic (ROC) curve analysis was performed to evaluate the prognostic performance of six nutritional and inflammatory indices (PNI, GNRI, HALP, SII, ALI, and CAR) for overall survival. Optimal cutoff values were determined using the Youden index.

Overall survival (OS) and progression-free survival (PFS) were analyzed using the Kaplan–Meier method and compared with the log-rank test. Univariate and multivariate Cox proportional hazards regression analyses were performed to identify independent prognostic factors. Because the PNI, GNRI, and HALP share common biological components, separate multivariable models were constructed for each index to minimize multicollinearity. Correlations among the PNI, GNRI, and HALP were assessed using Spearman rank correlation analysis. All statistical analyses were performed using IBM SPSS Statistics for MAC, Version 27.0 (IBM Corp., Armonk, NY, USA). A two-sided *p*-value < 0.05 was considered statistically significant.

## 3. Results

### 3.1. Patient Characteristics

A total of 192 patients with a confirmed diagnosis of DLBCL were included in the study. Baseline demographic and clinical characteristics are summarized in Table 1. The median age was 59 years (IQR, 22), and 63.5% of the patients were male. The mean ECOG performance status and IPI scores were 0.69 ± 0.89 and 2.37 ± 1.04, respectively. Most patients presented with advanced-stage disease (median Ann Arbor stage, 3), and 56.8% had extranodal involvement. The activated B-cell (ABC) subtype was identified in 57.8% of cases. R-CHOP was administered to 144 patients (75.0%), whereas 48 patients (25.0%) received R-EPOCH. Progressive disease was documented by post-treatment fluorodeoxyglucose positron emission tomography/computed tomography (FDG PET/CT) in 31 patients (16.1%) (Table 1). During a median follow-up of 36 months, 18 patients (9.4%) died. Baseline laboratory parameters are presented in Table 2.

### 3.2. ROC Analysis and Optimal Cut-Off Values

**Figure 1:**
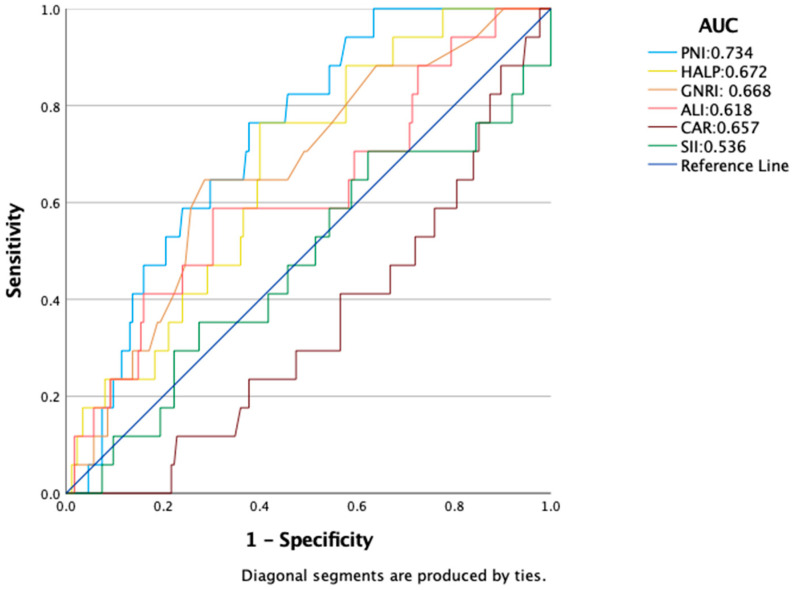
ROC Curves for All Six Nutritional-Inflammatory Indices.

ROC analysis was performed for all six candidate indices. Among them, the PNI achieved the highest predictive accuracy for overall survival (OS) (AUC = 0.734, 95% CI: 0.634–0.834, *p* = 0.001), followed by HALP (AUC = 0.671, 95% CI: 0.555–0.787, *p* = 0.020) and the GNRI (AUC = 0.668, 95% CI: 0.540–0.797, *p* = 0.022). The SII (AUC = 0.474, *p* = 0.727) and ALI (AUC = 0.618, *p* = 0.109) were not significantly associated with OS. CAR yielded an AUC below 0.50 (AUC = 0.346, *p* = 0.036), indicating an inverse association with survival outcome. Therefore, subsequent analyses focused on the PNI, GNRI, and HALP, which demonstrated the highest discriminatory performance (Table 3; Figure 1).

Using the Youden index, the optimal cutoff values were determined as ≤46.45 for the PNI (sensitivity 76.5%, specificity 62.3%), ≤46.91 for the GNRI (sensitivity 64.7%, specificity 71.4%), and ≤223.95 for HALP (sensitivity 76.5%, specificity 60.0%).

### 3.3. Association of Index Groups with Clinicopathological Characteristics

Based on the Youden-derived cutoff values, patients were classified into low and high groups for each index: GNRI (55 and 137 patients, respectively), PNI (79 and 113 patients, respectively), and HALP (80 and 112 patients, respectively). Associations between index groups and clinicopathological characteristics are presented in Table 4.

Low values of all three indices were associated with a higher IPI category (*p* = 0.002 for all comparisons), advanced Ann Arbor stage (*p* = 0.006, *p* = 0.021, and *p* = 0.040 for GNRI, PNI, and HALP, respectively), and elevated LDH levels (*p* < 0.001, *p* = 0.003, and *p* < 0.001, respectively). A low PNI and low HALP were also associated with poorer ECOG performance status (*p* = 0.035 and *p* = 0.027, respectively). In contrast, a low GNRI was associated with extranodal involvement (*p* = 0.036), whereas no significant association was observed for the PNI (*p* = 0.089) or HALP (*p* = 0.058). No significant differences were observed between index groups with respect to sex, age, cell of origin, or double-expressor status (Table 4).

### 3.4. Kaplan–Meier Survival Analysis

**Figure 2:**
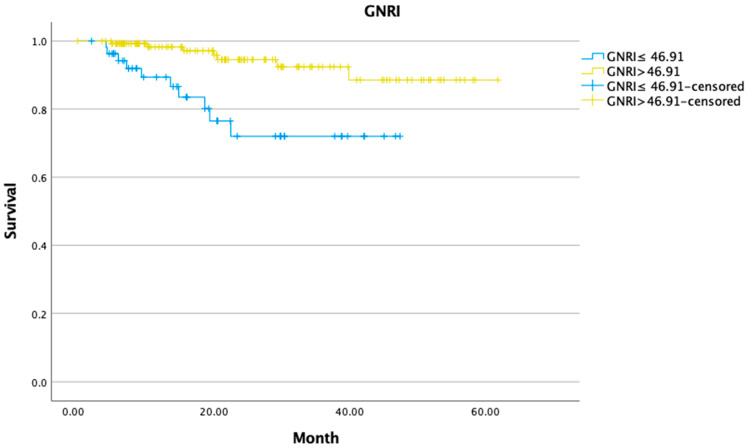
Kaplan–Meier overall survival curves classified according to the GNRI cutoff value (≤46.91 and >46.91).

**Figure 3:**
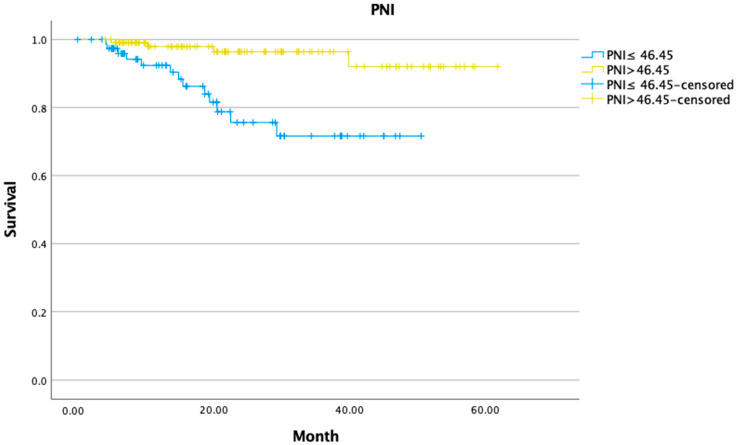
Kaplan–Meier overall survival curves classified according to the PNI threshold (≤46.45 and >46.45).

**Figure 4:**
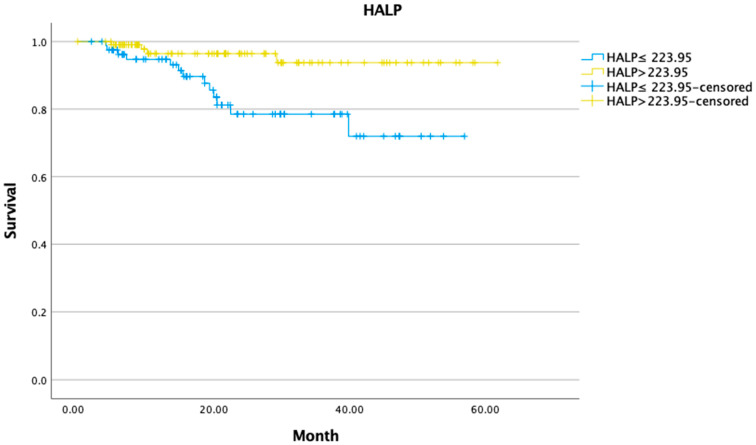
Kaplan–Meier OS curves classified by the HA‍LP cutoff value (≤223.95 vs. >223.95).

During the follow-up period, 18 patients (9.4%) died. The median overall survival (OS) was not reached in either group for any of the three indices. Patients with high GNRI values (>46.91) had significantly longer OS than those with low GNRI values (≤46.91), with mean survival times of 66.0 ± 1.4 and 49.6 ± 3.1 months, respectively (log-rank *p* = 0.001). Similarly, patients with a high PNI (>46.45) had longer OS than those with low PNI values, with mean survival times of 67.1 ± 1.2 and 52.8 ± 2.6 months, respectively (log-rank *p* = 0.001). Patients with high HALP values (>223.95) also demonstrated superior OS compared with those with low HALP values, with mean survival times of 64.9 ± 1.2 and 58.8 ± 2.8 months, respectively (log-rank *p* = 0.005). Kaplan–Meier survival curves are shown in Figure 2, Figure 3 and Figure 4. No significant differences in progression-free survival (PFS) were observed between the high- and low-index groups for the GNRI, PNI, or HALP (all *p* > 0.05).

### 3.5. Univariable and Multivariable Cox Regression Analysis for Survival Time

In univariable Cox regression analysis, high HALP (HR = 0.232, 95% CI: 0.076–0.712, *p* = 0.011), a high PNI (HR = 0.182, 95% CI: 0.059–0.559, *p* = 0.003), and a high GNRI (HR = 0.233, 95% CI: 0.088–0.612, *p* = 0.003) were significantly associated with improved overall survival (Table 5). IPI showed a trend toward significance at the overall level (*p* = 0.095), whereas the high-intermediate/high-risk IPI category reached borderline significance in pairwise comparison (HR = 4.720, *p* = 0.050). Sex was not significantly associated with overall survival in univariable analysis (*p* = 0.117). The treatment regimen (R-EPOCH vs. R-CHOP) was also evaluated and was not significantly associated with overall survival (HR = 0.391, 95% CI: 0.089–1.714, *p* = 0.213).

To account for shared biological components and potential multicollinearity among the PNI, GNRI, and HALP, three separate multivariable models were constructed, each adjusted for sex and IPI (Table 6). In all three models, the nutritional index remained an independent predictor of overall survival: PNI (HR = 0.216, 95% CI: 0.069–0.677, *p* = 0.009), HALP (HR = 0.276, 95% CI: 0.086–0.891, *p* = 0.031), and GNRI (HR = 0.294, 95% CI: 0.108–0.806, *p* = 0.011). Neither IPI nor sex remained independently associated with overall survival in any of the three models.

### 3.6. Univariable and Multivariable Cox Regression Analysis for PFS

No significant association was observed between PFS and a low PNI, a low GNRI, or low HALP in either univariate or multivariate Cox regression analyses. These findings suggest that the nutritional-inflammatory status measured by these indices is more strongly associated with long-term survival rather than early disease progression in this cohort.

### 3.7. Correlation Analysis Among Nutritional-Inflammatory Indices

Correlation analysis was performed to evaluate the relationships among the PNI, GNRI, and HALP. Significant positive correlations were observed between all three indices. The strongest correlation was observed between the PNI and HALP (Spearman’s rho = 0.792, *p* < 0.001), followed by the PNI and GNRI (rho = 0.733, *p* < 0.001). A moderate positive correlation was also observed between the GNRI and HALP (rho = 0.521, *p* < 0.001) (Table 7).

## 4. Discussion

In this single-center retrospective study, we evaluated six nutritional and inflammatory indices in a cohort of 192 patients with DLBCL treated with R-CHOP or R-EPOCH. Among these indices, the PNI demonstrated the highest predictive accuracy for overall survival (OS) (AUC = 0.734), followed by HALP and the GNRI. All three indices remained independently associated with OS in separate multivariable models adjusted for sex and IPI, supporting their potential value as readily available pre-treatment prognostic markers in DLBCL.

The prognostic role of the PNI in lymphoma is increasingly recognized. Go et al. demonstrated that a low PNI is an independent predictor of poorer OS in DLBCL patients treated with R-CHOP, consistent with our findings [16]. Similarly, Yu et al. reported that the PNI outperformed NLR and PLR as a prognostic tool in newly diagnosed DLBCL [15]. The biological basis of these associations lies in the two components of the PNI. Serum albumin reflects nutritional reserves, liver synthetic function, and the negative acute-phase response, whereas lymphocyte count reflects antitumor cellular immunity, including cytotoxic T-cell and NK-cell activity, which are critical for rituximab efficacy [22].

Low albumin and lymphopenia independently predict poor outcomes in DLBCL, and their combination in the PNI provides superior predictive value [23,24]. The relationship between these indices and nutritional status deserves particular attention. Albumin, which is incorporated into the PNI, GNRI, and HALP, reflects not only systemic inflammation but also nutritional reserve and protein-energy status. Likewise, lymphocyte depletion may be aggravated by malnutrition and is associated with impaired cellular immunity. Consequently, these indices may be viewed as composite markers of both nutritional status and host immune competence. Their prognostic significance may reflect the combined effects of nutritional reserve, systemic inflammation, and treatment tolerance.

Originally developed by Bouillanne and colleagues as a bedside nutritional screening tool for elderly bedridden patients, the GNRI combines body weight relative to ideal body weight and serum albumin [17]. Although its validity was initially established in surgical and geriatric oncology populations, accumulating evidence supports its prognostic value in hematologic malignancies [18]. Suzuki et al. demonstrated that a low GNRI is associated with poorer outcomes in multiple myeloma, and our findings suggest that this association may also extend to DLBCL [19]. Notably, a low GNRI was associated with extranodal involvement in our cohort (*p* = 0.036), whereas no significant association was observed for the PNI or HALP. Accurate baseline staging is essential when evaluating prognostic biomarkers in DLBCL because both Ann Arbor stage and extranodal involvement are major determinants of outcome. Contemporary staging relies heavily on whole-body FDG-PET/CT, which improves the detection of occult disease sites and provides a more accurate assessment of disease burden. Picardi et al. demonstrated that FDG-PET/contrast-enhanced CT can identify clinically occult disease involvement that may influence risk stratification and treatment planning [25]. In this context, the association between a low GNRI and extranodal involvement observed in our cohort may reflect the greater nutritional impairment accompanying more extensive disease burden and widespread lymphoma involvement.

An additional consideration is the evolving treatment landscape of DLBCL. More than half of our cohort consisted of ABC/non-GCB cases, a subgroup associated with poorer outcomes following conventional immunochemotherapy. Recent studies have reported improved outcomes with novel frontline approaches, including polatuzumab-containing regimens and intensified anthracycline-based strategies incorporating non-pegylated liposomal doxorubicin [26]. In this setting, the prognostic value of nutritional and inflammatory indices such as the PNI, GNRI, and HALP should be evaluated in patients receiving contemporary treatment regimens. Because these indices primarily reflect host nutritional and immune status rather than tumor-specific characteristics, their prognostic value may extend across different treatment approaches. However, this possibility requires confirmation in prospective studies.

HALP, a composite index that combines hemoglobin, albumin, lymphocyte, and platelet counts, reflects multiple aspects of the host immune-nutritional response. Hemoglobin indicates oxygen-carrying capacity and is an independent prognostic factor in lymphoma [1]. Platelet count reflects not only hemostatic function but also tumor-promoting interactions between platelets and circulating lymphoma cells [27].

Jiang et al. reported that a low HALP value independently predicts poor overall survival in non-small cell lung cancer, and subsequent studies have confirmed its prognostic significance in hepatocellular carcinoma and colorectal cancer [20,21]. Data on HALP in DLBCL remain limited. In our cohort, HALP demonstrated moderate discriminatory ability for overall survival (AUC = 0.671) and remained independently associated with overall survival in multivariable analysis (*p* = 0.031). These findings support the potential prognostic value of HALP in patients with DLBCL.

As expected, significant correlations were observed among the PNI, GNRI, and HALP. This overlap is biologically plausible because these indices share common components, particularly serum albumin and variables related to nutritional and immune status. Nevertheless, the correlations were not perfect, suggesting that each index captures partially distinct aspects of host physiology. While the PNI primarily reflects immunonutritional status through albumin and lymphocyte count, the GNRI additionally incorporates body weight, and HALP integrates hemoglobin and platelet parameters. Therefore, these indices should be regarded as complementary rather than interchangeable prognostic tools.

Unlike the PNI, GNRI, and HALP, the SII and ALI did not reach prognostic significance in our cohort (*p* = 0.727 and *p* = 0.109, respectively). This finding differs from several studies in solid tumors, where the SII has demonstrated prognostic value [28,29]. One possible explanation is the mechanism of action of rituximab, which targets CD20-positive B cells and exerts its antitumor effects through immune-mediated pathways, including antibody-dependent cellular cytotoxicity (ADCC) and complement-dependent cytotoxicity. In this setting, nutritional and immune parameters may better reflect patient outcomes than composite inflammatory indices. In addition, the relatively homogeneous treatment approach and the low number of deaths in our cohort may have reduced the ability of the SII and ALI to discriminate survival outcomes.

Notably, IPI did not retain independent significance in any of the three multivariable models. This finding may be related to the limited statistical power of the study, particularly given the small number of deaths (*n* = 18). No significant association was observed between progression-free survival and any of the three indices. This may be related to the relatively short follow-up period and the predominance of treatment-responsive disease in the cohort.

From a clinical perspective, our findings suggest that low PNI, GNRI, and HALP values are associated with inferior overall survival in patients with DLBCL. These readily available indices may help identify high-risk patients with impaired nutritional and immune reserves at diagnosis. Such patients may benefit from closer monitoring, comprehensive nutritional assessment, early dietitian involvement, optimization of supportive care, and careful management of treatment-related toxicities.

However, because of the retrospective observational design of this study, causality cannot be inferred. It also remains unclear whether interventions aimed at improving these indices would translate into better clinical outcomes. Prospective interventional studies are needed to determine whether correction of nutritional deficits can improve outcomes in patients with DLBCL.

This study has several limitations. First, its retrospective single-center design introduces the possibility of selection bias and limits generalizability. Second, the relatively small number of deaths (*n* = 18) reduced statistical power and may have contributed to the wide confidence intervals observed in some multivariable analyses. Third, residual confounding cannot be excluded despite the use of multivariable models. Factors such as comorbidities, nutritional interventions, socioeconomic status, frailty, and unmeasured inflammatory conditions may have influenced both biomarker values and clinical outcomes. In addition, some treatment heterogeneity was present because patients received either R-CHOP or R-EPOCH according to clinical indications. However, univariable Cox regression analysis demonstrated no significant association between treatment regimen and overall survival (HR = 0.391, 95% CI: 0.089–1.714, *p* = 0.213), suggesting that the prognostic value of the PNI, GNRI, and HALP was unlikely to be explained solely by treatment differences. Fourth, body weight data used for the GNRI calculation were obtained from available clinical records and may be subject to measurement variability. Fifth, longitudinal changes in the PNI, GNRI, and HALP during treatment were not evaluated. Finally, the absence of an external validation cohort limits the generalizability of our findings and underscores the need for confirmation in prospective multicenter studies.

## 5. Conclusions

This study suggests that the baseline PNI, GNRI, and HALP are independent prognostic markers of overall survival in patients with DLBCL treated with rituximab-based immunochemotherapy. Among the six indices evaluated, the PNI exhibited the highest discriminatory accuracy (AUC = 0.734), whereas the GNRI showed a unique association with extranodal involvement. These readily available and low-cost indices may complement IPI-based risk stratification and help identify patients at higher risk of adverse outcomes who may benefit from closer monitoring and comprehensive supportive care. Prospective multicenter studies are needed to validate these findings and to determine the optimal integration of nutritional and inflammatory indices into routine clinical assessment in DLBCL.

## Figures and Tables

**Table 1:** Demographic and Clinical Characteristics of Patients with DLBCL at Baseline.

Variable	Value
Number of patients, *n*	192
Age, median (IQR), years	59 (22)
Male sex, *n* (%)	122 (63.5%)
ECOG performance status, mean ± SD	0.69 ± 0.89
IPI score, mean ± SD	2.37 ± 1.04
Ann Arbor stage, median (IQR)	3 (2)
Extranodal involvement, *n* (%)	109 (56.8%)
Activated B-cell (ABC) subtype, *n* (%)	111 (57.8%)
Germinal center B-cell (GCB) subtype, *n* (%)	81 (42.2%)
R-CHOP treatment, *n* (%)	144 (75.0%)
R-EPOCH treatment, *n* (%)	48 (25.0%)
Progressive disease, *n* (%)	31 (16.1%)
Mortality, *n* (%)	18 (9.4%)

Data are presented as median (IQR) for non-normally distributed variables and mean ± SD for normally distributed variables.

**Table 2:** Baseline Laboratory Parameters.

Variable	Value
ESR (mm/h)	34 (IQR: 39)
CRP (mg/L)	16 (IQR: 44)
BMI (kg/m^2^)	25.7 (IQR: 6.3)
LDH (U/L)	259 (IQR: 168)
Albumin (g/dL)	4.0 (IQR: 0.9)
Hemoglobin (g/dL) *	11.9 ± 2.2
Neutrophils (cells/mm^3^)	4800 (IQR: 2800)
Lymphocytes (cells/mm^3^)	1430 (IQR: 1120)
Platelets (×10^3^/mm^3^) *	288 ± 114

Variables not following a normal distribution: median (IQR). * Variables following a normal distribution: mean ± SD. ESR, erythrocyte sedimentation rate; CRP, C-reactive protein; BMI, body mass index; LDH, lactate dehydrogenase.

**Table 3:** ROC Analysis of Six Nutritional and Inflammatory Markers for Overall Survival Prediction.

Index	AUC	95% CI	*p*-Value	Selected
PNI	0.734	0.634–0.834	**0.001**	✓
HALP	0.671	0.555–0.787	**0.020**	✓
GNRI	0.668	0.540–0.797	**0.022**	✓
ALI	0.618	0.471–0.764	0.109	—
CAR ^†^	0.346	0.222–0.470	0.036	—
SII	0.474	0.320–0.628	0.727	—

AUC, area under the curve; CI, confidence interval; PNI, prognostic nutritional index; HALP, hemoglobin-albumin-lymphocyte-platelet index; GNRI, geriatric nutritional risk index; ALI, advanced lung cancer inflammation index; CAR, C-reactive protein-albumin ratio; SII, systemic immune-inflammation index. ^†^ A CAR AUC < 0.5 indicates an inverse relationship (high CAR is associated with worse OS; reflected AUC = 0.654). ✓ indicates variables selected for further statistical analyses. Optimal cutoff values determined by the Youden index: PNI ≤ 46.45; GNRI ≤ 46.91; HALP ≤ 223.95.

**Table 4:** Association of the GNRI, PNI, and HALP Groups with Clinical and Pathological Characteristics.

Variable	GNRI			PNI			HALP		
	Low (*n* = 55)	High (*n* = 137)	*p*	Low (*n* = 79)	High (*n* = 113)	*p*	Low (*n* = 80)	High (*n* = 112)	*p*
Male sex	36 (65.5%)	86 (62.8%)	0.727	50 (63.3%)	72 (63.7%)	0.952	51 (63.8%)	71 (63.4%)	0.599
Age ≥ 60 years	30 (54.5%)	64 (46.7%)	0.206	45 (57.0%)	49 (43.4%)	0.064	45 (56.3%)	49 (43.8%)	0.203
High-intermediate/high IPI	36 (65.5%)	55 (40.1%)	**0.002**	49 (62.0%)	42 (37.2%)	**0.002**	50 (62.5%)	41 (36.6%)	**0.002**
ECOG performance status ≥ 2	13 (23.6%)	20 (14.6%)	0.133	19 (24.1%)	14 (12.4%)	**0.035**	20 (25.0%)	13 (11.6%)	**0.027**
Ann Arbor stage III–IV	46 (83.6%)	87 (63.5%)	**0.006**	62 (78.5%)	71 (62.8%)	**0.021**	64 (80.0%)	69 (61.6%)	**0.040**
Elevated LDH	42 (76.4%)	62 (45.3%)	<0.001	53 (67.1%)	51 (45.1%)	**0.003**	59 (73.8%)	45 (40.2%)	<0.001
Extranodal involvement ≥ 1 site	38 (69.1%)	72 (52.6%)	**0.036**	51 (64.6%)	59 (52.2%)	0.089	54 (67.5%)	56 (50.0%)	0.058
Double-expressor status	17 (30.9%)	34 (24.8%)	0.388	25 (31.6%)	26 (23.0%)	0.182	21 (26.3%)	30 (26.8%)	0.730
Activated B-cell (ABC) subtype	32 (58.2%)	79 (57.7%)	0.948	49 (62.0%)	62 (54.9%)	0.323	49 (61.3%)	62 (55.4%)	0.764

Pearson’s chi-square test. Bold *p*-values indicate statistical significance (*p* < 0.05). GNRI, Geriatric Nutritional Risk Index; PNI, Prognostic Nutritional Index; HALP, Hemoglobin–Albumin–Lymphocyte–Platelet score; IPI, International Prognostic Index; LDH, lactate dehydrogenase.

**Table 5:** Univariable Cox Regression Analysis for Overall Survival.

Variable	HR	95% CI	*p*-Value	Sig.
IPI (overall)	—	—	0.095	
IPI Low-Int vs. Low	1.637	0.274–9.800	0.589	
IPI High-Int vs. Low	4.720	0.998–22.318	**0.050**	
IPI High vs. Low	4.216	0.771–23.043	0.097	
Sex (female vs. male)	0.368	0.106–1.284	0.117	
Treatment (R-EPOCH vs. R-CHOP)	0.391	0.089–1.714	0.213	
HALP (>223.95 vs. ≤223.95)	0.232	0.076–0.712	**0.011**	✓
PNI (>46.45 vs. ≤46.45)	0.182	0.059–0.559	**0.003**	✓
GNRI (>46.91 vs. ≤46.91)	0.233	0.088–0.612	**0.003**	✓

HR, hazard ratio; CI, confidence interval. Treatment variable compares R-EPOCH with R-CHOP. The HR values for HALP, PNI, and GNRI use the high-index group as the reference (an HR <1 indicates better survival in the high-index group). ✓ indicates statistically significant variables (*p* < 0.05). Bolded *p*-values indicate statistical significance (*p* < 0.05).

**Table 6:** Multivariable Cox regression analyses for overall survival (three separate models).

Variable	HR	95% CI	*p*-Value	Sig.
Model 1: PNI + Sex + IPI (overall *p* = 0.004)				
Sex (female vs. male)	0.374	0.106–1.322	0.127	
IPI Low-Int vs. Low	1.139	0.187–6.945	0.888	
IPI High-Int vs. Low	2.997	0.619–14.512	0.173	
IPI High vs. Low	2.568	0.455–14.495	0.286	
PNI (>46.45 vs. ≤46.45)	0.216	0.069–0.677	**0.009**	✓
Model 2: HALP + Sex + IPI (overall *p* = 0.013)				
Sex (female vs. male)	0.364	0.103–1.281	0.115	
IPI Low-Int vs. Low	1.133	0.184–6.957	0.893	
IPI High-Int vs. Low	2.759	0.553–13.755	0.216	
IPI High vs. Low	2.272	0.387–13.328	0.363	
HALP (>223.95 vs. ≤223.95)	0.276	0.086–0.891	**0.031**	✓
Model 3: GNRI + Sex + IPI (overall *p* = 0.003)				
Sex (female vs. male)	0.396	0.113–1.382	0.146	
IPI Low-Int vs. Low	1.117	0.180–6.920	0.906	
IPI High-Int vs. Low	2.918	0.586–14.545	0.191	
IPI High vs. Low	2.720	0.472–15.668	0.263	
GNRI (>46.91 vs. ≤46.91)	0.294	0.108–0.806	**0.011**	✓

To avoid multicollinearity, separate multivariate models were constructed for each index (all three indices share albumin and/or lymphocyte count as common components). HR: hazard ratio; CI: confidence interval. ✓ indicates statistically significant variables (*p* < 0.05). Bold *p*-values: *p* < 0.05.

**Table 7:** Correlation analysis among PNI, GNRI, and HALP.

Variable	PNI	GNRI	HALP
PNI	1.000	0.733 *	0.792
GNRI	0.733 *	1.000	0.521
HALP	0.792 *	0.521	1.000

Spearman correlation coefficient, *p* < 0.001 for all comparisons. * Indicates a statistically significant correlation (*p* < 0.001).

## Data Availability

The data supporting the findings of this study are available from the corresponding author upon reasonable request. However, due to patient confidentiality and privacy regulations, the data are not publicly available and can only be shared when necessary and under appropriate ethical and institutional approvals.
